# The Dental Register of the West

**Published:** 1847-12

**Authors:** 


### PART II.—BIBLIOGRAPHICAL NOTICES.

### ARTICLE IX.

*The Dental Register of the West.* Published quarterly, by order of the Mississippi Valley Association of Dental Surgeons. Edited by James Taylor, M. D., D. D. S., of Cincinnati, O., and B. B. Brown, M. D., of St. Louis, Mo. (In exchange.)

The first number of this publication is on our table; it shows a commendable degree of interest for improvement among the Dentists of the West. Its contents appear to be well made up, and mostly of original matter, on subjects directly pertaining to the theory and practice of Dental Surgery. We are gratified to see the zeal with which our friends of the dental profession are taking hold of the subject of reform, with a view of improving the practice and elevating the standard of professional acquirements. We observe that they are disposed to discountenance quackery by disclaiming fellowship with those who are guilty of it. As they have now two colleges for educating dentists, one in the east at Baltimore, and one in the west at Cincinnati, which confer the degree of Doctor of Dental Surgery upon those who comply with the requirements, and come up to the standard, may we not hope soon to see the possession of this degree a passport to public confidence? E.

